# Santamarine Inhibits NF-*κ*B Activation and Induces Mitochondrial Apoptosis in A549 Lung Adenocarcinoma Cells via Oxidative Stress

**DOI:** 10.1155/2017/4734127

**Published:** 2017-10-02

**Authors:** Xuefeng Wu, Hua Zhu, Jingzhe Yan, Muhammad Khan, Xiuyan Yu

**Affiliations:** ^1^Department of Clinical Laboratory, Jilin Provincial Tumor Hospital, Changchun 130012, China; ^2^Department of Abdominal Oncosurgery-2, Jilin Provincial Tumor Hospital, Changchun 130012, China; ^3^College of Basic Medical Sciences, Dalian Medical University, Dalian, Liaoning 116044, China

## Abstract

Santamarine (STM), a sesquiterpene lactone component of* Magnolia grandiflora* and* Ambrosia confertiflora*, has been shown to possess antimicrobial, antifungal, antibacterial, anti-inflammatory, and anticancer activities. However, no study has yet been conducted to investigate the molecular mechanism of STM-mediated anticancer activity. In the present study, we found that STM inhibits growth and induces apoptosis in A549 lung adenocarcinoma cells through induction of oxidative stress. STM induces oxidative stress by promoting reactive oxygen species (ROS) generation, depleting intracellular glutathione (GSH), and inhibiting thioredoxin reductase (TrxR) activity in a dose-dependent manner. Further mechanistic study demonstrated that STM induces apoptosis by modulation of Bax/Bcl-2 expressions, disruption of mitochondrial membrane potential, activation of caspase-3, and cleavage of PARP in a dose-dependent manner. Moreover, STM inhibited the constitutive and inducible translocation of NF-*κ*Bp65 into the nucleus. IKK-16 (I-*κ*B kinase inhibitor) augmented the STM-induced apoptosis, indicating that STM induces apoptosis in A549 cells at least in part through NF-*κ*B inhibition. Finally, STM-induced apoptosis and expressions of apoptosis regulators were effectively inhibited by thiol antioxidant N-acetyl-L-cysteine (NAC), indicating that STM exerts its anticancer effects mainly through oxidative stress. To the best of our knowledge, this is the first report providing evidence of anticancer activity and molecular mechanism of STM.

## 1. Introduction

Lung cancer is the most prevalent malignancy and leading cause of cancer-related deaths both in males and in females worldwide, with approximately 1.4 million deaths annually [1]. Lung cancer can be broadly categorized into two main types: small-cell lung cancer (SCLC) and non-small-cell lung cancer (NSCLC) [2]. NSCLC is the most common cancer which represents about 85–90% of all lung cancer cases [3]. Despite concerted efforts to improve the current therapies, the prognosis of NSCLC remains very poor with a 5-year survival rate of about 15% [1]. In the past few decades, research scientists have mainly focused on the development of highly targeted anticancer drugs by exploiting the genetic differences between cancer cells and normal cells. The epidermal growth factor receptor (EGFR) has been frequently found to be mutated and overexpressed in NSCLC. At present, the most effective chemotherapeutic drugs for NSCLC are gefitinib, erlotinib, and afatinib, which are highly targeted EGFR-directed tyrosine kinase inhibitors (TKIs) [4]. However, genetic instability, aberrant activation of signaling pathways, and development of secondary drug resistance have become the major limitations of such drugs [5]. Exploring novel therapeutic agents with promising anticancer activity and their molecular mechanisms is, therefore, necessary for improving the outcome of NSCLC treatment.

Aerobic glycolysis, higher ROS level, and uncontrolled proliferation are the hallmarks of cancer cells [6]. Targeting the hallmarks of cancer is a rational approach for next-generation cancer therapy. It is well established now that cancer cells preferentially use aerobic glycolysis for their energy demand and higher growth rate. Due to the altered metabolism, cancer cells produce higher levels of ROS as compared to normal cells [7]. Higher ROS levels play an important role in cancer cell survival and proliferation. Recent research has shown that this unique biochemical property of cancer cells can be exploited for therapeutic benefits. ROS act as double-edged sword in living cells. At a low level, ROS act as signaling molecules and play a vital role in various biological processes including cell survival, proliferation, differentiation, and gene expression; at a higher level, however, ROS exert oxidative stress and induce cell death through various signaling pathways [8, 9]. Killing cancer cells by exploiting ROS metabolism in cancer cells with ROS-inducing agents has been shown to be feasible in various* in vitro* and* in vivo* experimental models [9–11]. ROS-based anticancer drug development strategy holds promise to kill heterogonous cancer cells effectively as it can be applied more broadly against various human cancers of multiple origins irrespective of their genotype and is less likely to suffer from drug resistance. Therefore, it is necessary now to explore the novel therapeutic agents that target ROS metabolism of cancer cells for the future development of highly effective anticancer drugs.

In the present study, we have screened a small molecules compound library consisting of 300 natural compounds using A549 lung cancer cells, in the presence or absence of NAC, a ROS scavenger. This screening strategy allowed us to identify novel bioactive compounds targeting ROS metabolism. Santamarine (STM) was identified as a novel ROS-based anticancer compound during the screening process. STM is a sesquiterpene lactone compound present in various plant species such as* Inula helenium*,* Inula japonica *[12],* Magnolia grandiflora *L. [13],* Ambrosia confertiflora* [14], and* Saussurea lappa *[15]. It has been reported to exhibit mycobactericidal effects [14]. It has also been shown to inhibit the growth of human gynecologic cancer cells [12]. However, no mechanistic study has yet been done to explore the STM-induced anticancer activity. Here, we provide evidence for the first time that STM exerts an anticancer activity in A549 lung adenocarcinoma cells by inducing oxidative stress.

## 2. Materials and Methods

### 2.1. Reagents and Antibodies

STM (purity > 98%) was purchased from Tauto Biotech (Shanghai, China). Dulbecco's Modified Eagle's Medium (DMEM) and fetal bovine serum (FBS) were obtained from Gibco (Eggenstein, Germany). Penicillin and streptomycin were purchased from Solarbio Co., Ltd. (Beijing, China). Annexin V-FITC apoptosis detection kit, ROS assay kit, mitochondrial membrane potential assay kit with JC-1, GSH/GSSG assay kit, and NAC were obtained from Beyotime (Nanjing, China). Propidium iodide (PI), calcein AM, dimethylsulfoxide (DMSO), protease inhibitor cocktail, 3-(4,5-dimethylthiazol-2-yl)-2,5-diphenyltetrazolium bromide (MTT), and phenylmethylsulfonyl fluoride (PMSF) were purchased from Sigma-Aldrich (St. Louis, MO). Thioredoxin reductase assay kit was obtained from Abcam. Human TNF-a was purchased from Sino Biological Technology (Beijing, China). The primary antibodies for Bax, Bcl-2, GAPDH, and Lamin B1 were obtained from Proteintech (Wuhan, China). Primary antibodies for cleaved caspase-3, cleaved PARP, and NF-*κ*Bp65 were obtained from Cell Signaling Technology (Beverly, MA). Horseradish peroxidase- (HRP-) conjugated secondary antibodies (goat anti-rabbit, goat anti-mouse) were obtained from Sigma.

### 2.2. Cell Culture and Treatment

Human A549 and NCI-H1650 NSCLC cells and NL-20 normal lung cells were obtained from American Type Culture Collection (Manassas, VA) and cultured in DMEM supplemented with 10% FBS, 100 units/mL penicillin, and 100 *μ*g/mL streptomycin at 37°C with 5% CO_2_ in a humidified atmosphere. Cells were treated with STM dissolved in DMSO with a final DMSO concentration of 0.5%. DMSO treated cells were used as control.

### 2.3. Determination of Cell Viability by MTT Assay

Human lung cancer cells (A549 and NCI-H1650) and normal lung cells (NL-20) were incubated with the indicated concentrations of STM for 24 h in 96-well plates and cell viability was determined by MTT assay as described by us previously [16]. Drug treated cells were then incubated with 10 *μ*L MTT reagent (5 mg/mL) at 37°C for 4 h. Subsequently, the medium was removed and 150 *μ*L DMSO was added to dissolve farmazan crystals. The absorbance was measured at 570 nm (*A*_570_) by a microplate reader (Synergy Neo HTS multimode microplate reader, BioTek) and the percentage of cell viability was calculated as follows:

(1) Cell  viability%=A570  sample−A570  blankA570  control−A570  blank×100.

### 2.4. Observation of Morphological Changes of Cells

Human NSCLC (A549 and NCI-H1650) cells and normal lung cells (NL-20) were treated with 0, 40, and 60 *μ*M of STM with or without 3 mM NAC for 24 h. Following treatment, cell morphological changes were observed under a phase-contrast microscope (Leica, DMIL LED) and photographed.

### 2.5. Live/Dead Assay

A549, NCI-H1650, and NL-20 cells were treated with 0, 40, and 60 *μ*M of STM in the presence or absence of 3 mM NAC for 24 h. Live/dead assay was performed to quantify live and dead cells as described previously [11]. Briefly, cells were harvested, washed with phosphate-buffered saline (PBS), and incubated with 2 *μ*M calcein AM and 4 *μ*M PI for 15 min in the dark at room temperature. After washing, cells were resuspended in PBS and analyzed for the fluorescence of calcein and PI under a fluorescence microscope (Leica, DMI 4000B). Finally, three different areas were randomly selected and 100 cells were counted microscopically from each area for the percentage of live and dead cells.

### 2.6. Apoptosis Assay

The apoptotic effect of STM was evaluated by Annexin V-FITC/PI double staining kit (Beyotime). Briefly, A549 cells were treated with 0, 40, and 60 *μ*M of STM in the presence or absence of 3 mM NAC for 24 h. After drug treatment, the cells were harvested, washed with PBS, resuspended in binding buffer, and incubated with 5 *μ*L Annexin V and 10 *μ*L PI in the dark for 15 min according to the kit instructions. The samples were analyzed by flow cytometry (BD Accuri C^6^) for the percentage of apoptotic cells.

### 2.7. Measurement of ROS Generation

The intracellular changes in ROS generation were evaluated by staining the cells with 2′,7′-dichlorofluorescein diacetate (DCFH-DA) according to the kit instructions (Beyotime). The fluorescent dye DCFH-DA being cell membrane permeable enters the cells where it is converted into a cell membrane impermeable nonfluorescent compound DCFH by the activity of intracellular esterases. Oxidation of DCFH by ROS produces a highly fluorescent DCF. The fluorescence intensity of DCF inside the cells is directly proportional to the amount of peroxide produced. Briefly, A549 cells were treated with STM in a dose- and time-dependent manner. Following drug treatment, the cells were harvested and incubated with DCFH-DA for 30 min in the dark. After washing with DMEM 3 times, DCF fluorescence was measured at an excitation wavelength of 488 nm and an emission wavelength of 525 nm by a fluorescence microplate reader (Synergy Neo HTS multimode microplate reader, BioTek).

### 2.8. Measurement of GSH/GSSG Ratio

A549 cells were treated with 0, 40, and 60 *μ*M of STM in the presence or absence of 3 mM NAC for 24 h. The cells were collected and intracellular GSH/GSSG ratio was measured spectrophotometrically using GSH/GSSG assay kit (Beyotime) according to kit instructions.

### 2.9. Measurement of Mitochondrial Membrane Potential (MMP)

The effect of STM on MMP was evaluated by MMP assay kit (Beyotime). Briefly, A549 cells were incubated with 0, 40, and 60 *μ*M of STM in the presence or absence of 3 mM NAC for 24 h. After drug treatment, the cells were washed with PBS and stained with JC-1 fluorescent probe according to the kit instructions for 20 min in the dark. The cells were washed and fluorescence distribution of JC-1 monomers (green fluorescence) and J-aggregates (red fluorescence) was measured by a fluorescence microplate reader (Synergy Neo HTS multimode microplate reader, BioTek). The MMP was calculated by a decrease in red/green fluorescence intensity ratio.

### 2.10. Immunoblotting

Following drug treatment, adherent and floating cells were collected and washed with cold PBS and whole cell lysates were prepared using cell lysis buffer containing 20 mM Tris (pH 7.5), 150 mM NaCl, 1% Triton X-100, 50 mM NaF, 0.1 mM PMSF, sodium pyrophosphate, *β*-glycerophosphate, EDTA, Na3VO4, and Leupeptin (Beyotime Biotechnology) on ice for 30 min. Nuclear proteins were extracted using ProteinExt™ mammalian nuclear and cytoplasmic extraction kit (TransBionovo, Beijing, China). The protein concentration was determined by Enhanced BCA protein assay kit (Beyotime). A total of 20 *μ*g protein was separated on 10% sodium dodecylsulfate polyacrylamide gel electrophoresis (SDS-PAGE) and transferred to polyvinylidene difluoride (PVDF) membrane. After blocking with 5% nonfat milk, the membranes were incubated with Bax (1 : 500), Bcl-2 (1 : 500), cleaved caspase-3 (1 : 1000), cleaved PARP (1 : 1000), p65 (1 : 1000), GAPDH (1 : 1000), and Lamin B1 (1 : 500) antibodies overnight at 4°C. The membranes were washed with Tris-buffered saline-Tween (TBST) solution three times and incubated with HRP-conjugated goat anti-rabbit IgG (1 : 5000) or goat anti-mouse IgG (1 : 5000) secondary antibodies for 1 h at room temperature. The membranes were washed with TBST 3 times and immunoreactive bands were detected by Immobilon Western Chemiluminescent HRP Substrate (Millipore, Billerica, MA) and chemiluminescence images were obtained using MicroChemi 4.2 imaging system (DNR Bio-Imaging Systems).

### 2.11. Statistical Analysis

The results are expressed as mean ± standard error mean (SEM) of 3 different experiments and statistically compared with the control group or within the groups using one-way ANOVA followed by Tukey's Multiple Comparison Test.

## 3. Results

### 3.1. STM Inhibits Growth and Induces Cell Death in NSCLC Cells

**Figure 1:**
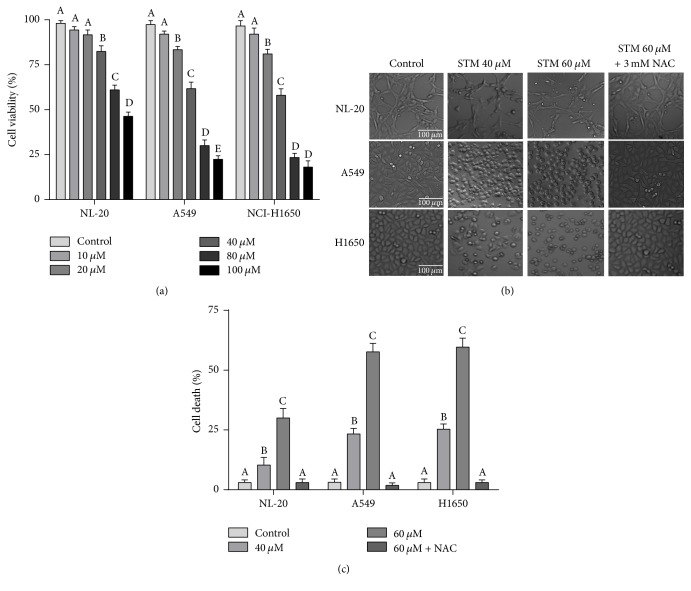
Effect of STM on proliferation and cell death. (a) NL-20, A549, and NCI-H1650 cells were treated with the indicated concentrations of STM for 24 h and cell proliferation was determined by the MTT assay. Data are expressed as mean ± SEM of three different experiments. Columns not sharing the same superscript letter differ significantly (*P* < 0.05). (b) NL-20, A549, and NCI-H1650 cells were treated with the indicated concentrations of STM in the presence or absence of 3 mM NAC for 24 h. Cells' morphological changes were observed under a phase-contrast microscope and images were captured. (c) NL-20, A549, and NCI-H1650 cells were treated with the indicated concentrations of STM for 24 h and live and dead cells were quantified using live/dead assay. Pretreatment of cells with NAC reversed STM-induced death in A549 cells. Data are expressed as mean ± SEM (*n* = 3). Columns not sharing the same superscript letters differ significantly (*P* < 0.05).

The cytotoxicity of STM against NSCLC was evaluated using A549 and NCI-H1650 cells. STM treatment for 24 h effectively inhibited the growth of A549 and NCI-H1650 cells* in vitro* in a dose-dependent manner as shown in Figure 1(a). The IC_50_ values were found to be around 45 and 43 *μ*M at 24 h drug treatment against A549 and H1650 cells, respectively. However, the growth inhibitory effect of STM against NL-20 normal lung cells was remarkably low with IC_50_ value around 85 *μ*M (Figure 1(a)). For further mechanistic study, 40 and 60 *μ*M concentrations were selected. The cytotoxic effect of STM was further evaluated by microscopic analysis of cell morphology. As shown in Figure 1(b), STM induced severe morphological changes characteristically associated with cell death such as junction breaks and rounding up of cells both in A549 and in NCI-H1650 cells in a dose-dependent manner. In contrast to lung cancer cells, STM was found to be less toxic against NL-20 normal lung cells as evident from microscopic observation of morphological changes (Figure 1(b)). Pretreatment of cells with 3 mM NAC completely reversed the cytotoxic effect of STM, indicating that STM-induced morphological changes are oxidative-stress-dependent. To further confirm and quantify STM-induced cell death, we performed live/dead assay. As shown in Figure 1(c), STM induced cell death both in A549 and in NCI-H1650 cells in a similar fashion in a dose-dependent manner, while NL-20 cells were found to be quite resistant against STM at the same concentrations and time point. In line with the data presented in Figure 1(b), pretreatment of cells with NAC completely inhibited the toxicity of STM as shown in Figure 1(c). The data suggest that STM induces cell death in lung cancer cells by promoting oxidative stress. For further mechanistic study, A549 cell line was used as a model cell line of NSCLC in this study.

### 3.2. STM Induces Apoptotic Cell Death in A549 Cells

**Figure 2:**
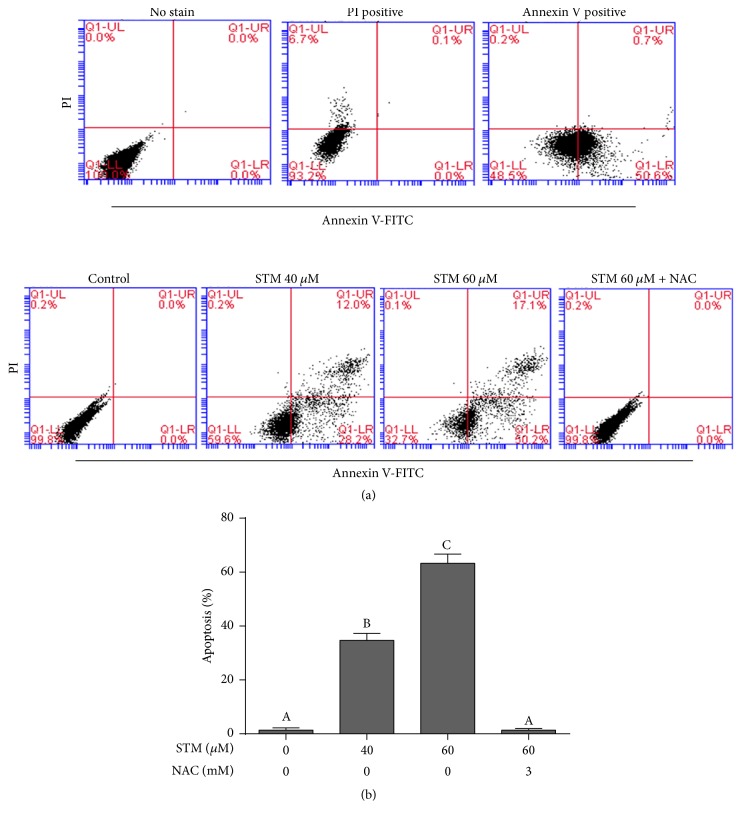
STM induces apoptosis in A549 cells. (a) A549 cells were treated with 0 and 40 *μ*M STM for 24 h. The cells treated with 40 *μ*M STM were stained either with PI or with Annexin V-FITC, while untreated cells were not stained with either probe. The samples were run on flow cytometry for compensation controls (upper). A549 cells were treated with the indicated concentration of STM in the presence or absence of 3 mM NAC for 24 h. Cells were stained with Annexin V/PI and apoptosis was determined by flow cytometry (lower). (b) Statistical analysis of data from (a). Columns not sharing the same superscript letters within the same group differ significantly (*P* < 0.05).

Live/dead assay can be used to quantify live and dead cells. To characterize the nature of cell death induced by STM, we performed the apoptosis assay by staining the cells with Annexin V-FITC/PI staining. The data demonstrated that STM induces apoptotic cell death in a dose-dependent manner (Figure 2). STM-induced apoptosis was reversed by pretreatment of cells with 3 mM NAC as shown in Figure 2.

### 3.3. STM Induces ROS Generation in A549 Cells

**Figure 3:**
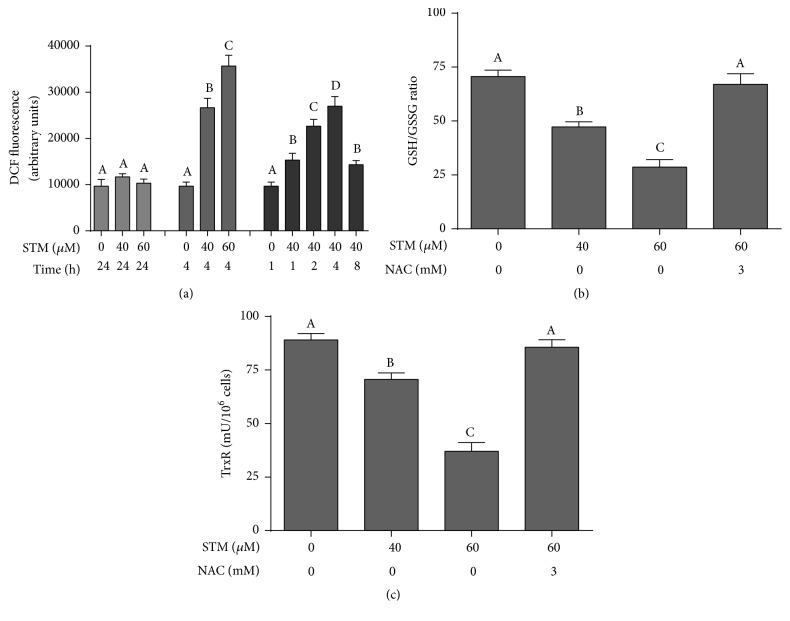
STM induces oxidative stress in A549 cells. (a) A549 cells were treated with STM in a dose- and time-dependent manner as indicated and intracellular ROS generation was measured by staining the cells with DCFH-DA. (b) A549 cells were treated with STM for 24 h in the presence or absence of NAC and intracellular GSH/GSSG ratio was measured according to the kit instructions. (c) A549 cells were treated with STM for 24 h in the presence or absence of NAC and TrxR activity was measured according to the kit instructions. Data (from (a), (b), and (c)) are expressed as mean ± SEM of three different experiments. Columns not sharing the same superscript letters within the same group differ significantly (*P* < 0.05).

As shown in Figures 2 and 3, NAC (ROS scavenger as well as GSH precursor) effectively abrogated the STM-induced cell death in A549 cells. Therefore, we measured the intracellular ROS level in control and STM treated cells. No change in intracellular ROS level was detected after 24 h drug treatment. Therefore, we measured the level of ROS in a time-dependent manner. The data showed that STM induces ROS generation in a time-dependent manner. As shown in Figure 3, STM started to increase ROS generation as early as 1 h of treatment which reached its maximum level at 4 h and then started to decrease at 8 h. Next, we measured the level of ROS in a dose-dependent manner at 4 h drug exposure. The data showed that STM increased ROS generation in a dose-dependent manner at 4 h drug treatment (Figure 3(a)).

### 3.4. STM Decreases GSH/GSSG Ratio in A549 Cells

Intracellular GSH is the major antioxidant that prevents cells from ROS-mediated toxicity. Depletion of intracellular GSH level is an early hallmark in ROS-mediated apoptosis in multiple cancer cells [16]. GSH/GSSG ratio is critical for cellular redox potential. A decrease in GSH/GSSG ratio is a sensitive indicator of oxidative stress. Therefore, we measured GSH/GSSG ratio. STM decreased intracellular GSH/GSSG ratio in a dose-dependent manner (Figure 3(b)). This GSH depletion was effectively inhibited by NAC pretreatment.

### 3.5. STM Inhibits Intracellular Thioredoxin Reductase (TrxR) Activity

Thioredoxin (Trx) is another important antioxidant system of cells that protects the cells from the damaging effect of high ROS and maintains redox status of the cells. TrxR is an important component of the thioredoxin (Trx) system [11]. Therefore, we wonder if STM could inhibit TrxR activity in A549 cells. As shown in Figure 3(c), STM significantly attenuated the activity of TrxR in a dose-dependent manner. Pretreatment of cells with 3 mM NAC completely reversed the effect of STM on TrxR activity.

### 3.6. STM Induces Oxidative-Stress-Dependent Mitochondrial Apoptosis in A549 Cells

**Figure 4:**
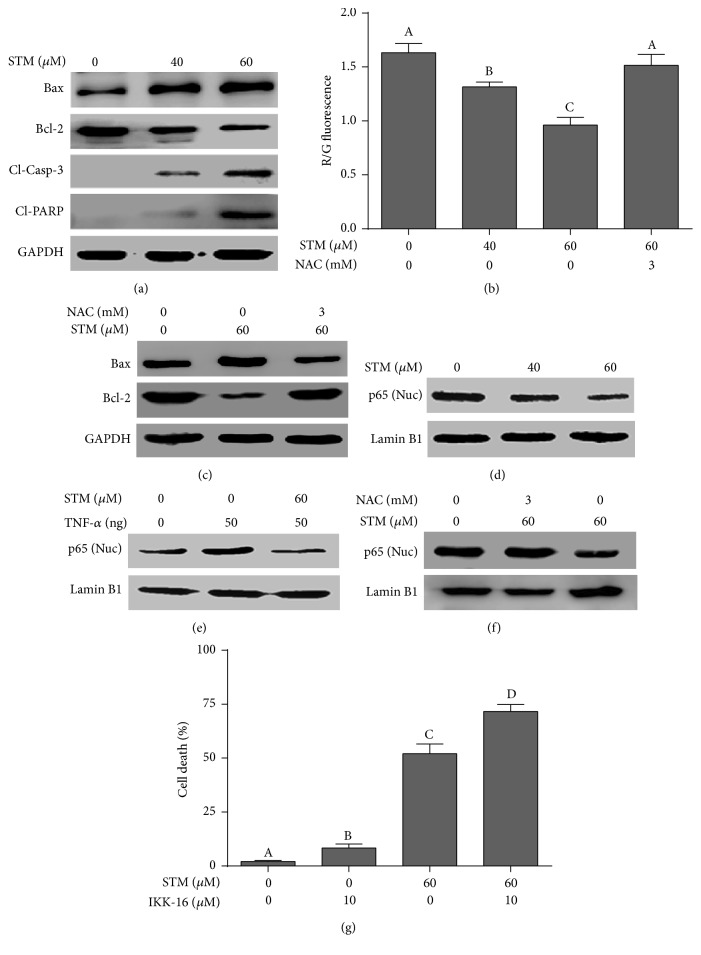
Effect of STM on apoptosis regulators. (a) A549 cells were treated with the indicated concentrations of STM for 24 h. Total cell lysates were extracted and subjected to western blots for the expression of Bax, Bcl-2, cleaved caspase-3, and cleaved PARP. GAPDH was used as a loading control. (b) A549 cells were treated with the indicated concentrations of STM in the presence or absence of NAC and MMP was measured using JC-1 kit according to the manufacturer's instructions. Columns not sharing the same superscript letters differ significantly (*P* < 0.05). (c) A549 cells were treated with STM in the presence or absence of NAC and cell lysates were extracted and subjected to western blots for the expression of Bax and Bcl-2. Pretreatment of cells with NAC reversed the STM-mediated proteins expression. (d, e, f) Cells were treated with STM in the presence or absence of NAC and TNF-*α* for 24 h. The expression of NF-*κ*Bp65 was measured in nuclear extracts. Lamin B1 was used as a loading control. NAC abolished the inhibitory effect of STM on NF-*κ*Bp65. (g) Cells were treated with IKK-16 either alone or in combination with STM for 24 h and cell death was determined by live/dead assay. Data are expressed as mean ± SEM of three different experiments. Columns not sharing the same superscript letters differ significantly (*P* < 0.05).

The above set of data indicates clearly that STM induces oxidative stress in A549 cells. Mitochondria are the source as well as target of ROS. Higher oxidative stress has been shown to induce apoptosis through mitochondrial pathway. Bcl-2 family protein modulation and mitochondrial membrane potential dissipation are characteristic features of mitochondrial apoptosis [16]. Therefore, we measured the expression of Bcl-2 and Bax by western blot. The data showed that STM decreased the expression of antiapoptotic Bcl-2 protein and increased the expression of proapoptotic Bax protein in a dose-dependent manner (Figure 4(a)). Next, we measured mitochondrial membrane potential. As shown in Figure 4(b), STM dissipated MMP in a dose-dependent manner. The effect of STM on Bcl-2 family proteins modulation and MMP disruption was completely abolished when cells were pretreated with 3 mM NAC. The data provide clear evidence that STM-induced apoptosis events are mainly mediated through oxidative stress. Cleavage of caspase-3 and PARP is considered the hallmark of apoptosis. Therefore, we measured the expressions of cleaved caspase-3 and cleaved PARP. The data demonstrated that STM dose-dependently increased the expression of cleaved caspase-3 and cleaved PARP in A549 cells (Figure 4).

### 3.7. STM Inhibits Constitutive and TNF-*α*-Induced Activation of NF-*κ*Bp65 in A549 Cells

**Figure 5:**
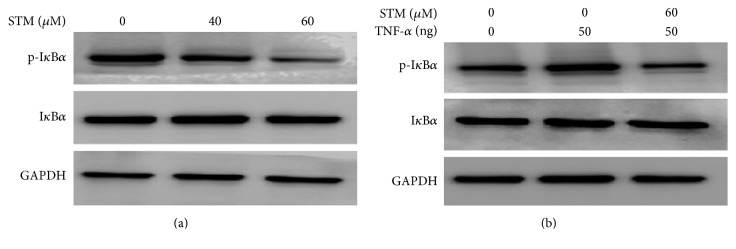
STM inhibits phosphorylation of I*κ*B-*α* in A549 cells. (a) Cells were treated with STM as indicated for 24 h. Total cell lysates were extracted and subjected to western blot for the expression and phosphorylation of I*κ*B-*α*. GAPDH was used as a loading control. (b) Cells were incubated with or without 60 *μ*M STM for 1 h and then stimulated with TNF-*α* for 1 h. Total cell lysates were extracted and subjected to western blot for the expression and phosphorylation of I*κ*B-*α*.

Sesquiterpene lactone compounds have been shown to inhibit NF-*κ*B activation in various cancer cells [17]. Therefore, we asked if STM could inhibit NF-*κ*B activation in A549 cells. For this, we measured the expression of NF-*κ*Bp65 in nuclear extracts. The data showed that STM inhibited the translocation of NF-*κ*Bp65 into the nucleus in a dose-dependent fashion (Figure 4(d)). In addition to measuring the effect on constitutive NF-*κ*B activation, we determine the effect of STM on TNF-*α*-induced translocation of NF-*κ*Bp65 into the nucleus. As shown in Figure 4(e), STM inhibited the TNF-*α*-induced translocation of NF-*κ*Bp65 into the nucleus. Next, we raised the question of whether STM-induced inhibition of NF-*κ*B activation is also mediated through oxidative stress. For this, we exposed the cells to STM in the presence or absence of NAC for the indicated time points and the expression of NF-*κ*Bp65 was measured in nuclear extracts by western blot. We found that NAC reversed the inhibitory effect of STM on NF-*κ*Bp65 translocation into the nucleus (Figure 4(f)). The data indicate clearly that STM inhibits NF-*κ*B activation through a process that involves oxidative stress. Finally, we were interested to know if NF-*κ*B inhibition is involved in STM-induced apoptosis in A549 cells. To answer this question, we treated the cells with IKK-16 either alone or in combination with STM for 24 h and cell death was determined by live/dead assay. The data demonstrated that IKK-16 treatment in combination with STM significantly increased the toxicity of STM as compared to STM treatment alone, showing that NF-*κ*B inhibition plays an important role in STM-induced apoptosis in A549 cells. To probe the possible mechanism of STM-mediated NF-*κ*B inhibition, we measured the expression and phosphorylation of I*κ*B-*α*. The data demonstrated that STM treatment inhibited the phosphorylation of I*κ*B-*α* without affecting its expression in a dose-dependent manner (Figure 5).

## 4. Discussion

It has become an established phenomenon now that cancer cells contain a higher level of ROS and are in the state of higher oxidative stress compared to normal cells [18, 19]. Higher oxidative stress is an adaptation of cancer cells, which plays an important role in cancer cell survival and proliferation [9]. Modern research has provided convincing evidence that this biochemical adaptation of cancer cells can be exploited for therapeutic benefits. Higher oxidative stress to toxic threshold can induce severe damage to lipids, proteins, and DNA, ultimately leading to cell death. As cancer cells contain higher oxidative stress compared to normal cells, the toxic threshold can be easily achieved by exposing cancer cells to phytochemicals targeting ROS metabolism and/or inhibiting the antioxidant system of the cells [9, 18, 20]. In the present study, we identified a novel natural bioactive compound “STM” as an oxidative stress inducer in A549 lung cancer cells using a ROS-based screening strategy.

STM is a sesquiterpene lactone compound present in various plant species such as* Magnolia grandiflora *L.,* Ambrosia confertiflora*,* Inula helenium*,* Inula japonica*, and* Saussurea lappa *[12–15]. Sesquiterpene lactones are 15-C compounds containing an *α*-methylene-*γ*-lactone moiety. The *α*-methylene-*γ*-lactone moiety of sesquiterpene lactones acts as an alkylating agent and can interact with sulfhydryl group of enzymes, transcriptional factors, and free intracellular glutathione (GSH) by means of Michael-type conjugation. Due to the high reactivity of *α*-methylene-*γ*-lactone moiety, sesquiterpene lactones can simultaneously interact with various signaling pathways and affect multiple targets in cancer cells [17, 21]. Recent research has shown that sesquiterpene lactones can effectively disrupt the redox homeostasis of cancer cells by depleting the antioxidant system of cells and increasing intracellular ROS generation [11, 16, 22]. GSH and thioredoxin are the two major antioxidant systems of the cells involved in the maintenance of the redox status of cells by detoxifying ROS and repairing ROS-induced damage to proteins and nucleic acids [11, 19]. In the present study, STM induced oxidative stress in A549 cancer cells by increasing ROS generation and decreasing intracellular GSH and thioredoxin reductase (TrxR) activity. The induction of oxidative stress by STM is further supported by other studies where alantolactone, a sesquiterpene lactone compound that shares the same *α*-methylene-*γ*-lactone moiety with STM, also induces oxidative stress in various cancer cells by GSH depletion, ROS generation, and inhibition of TrxR activity [16, 22, 23].

Once oxidative stress is induced, it acts as a second messenger to activate diverse redox-sensitive signaling cascades including mitochondrial-dependent apoptotic cascade through interaction with Bcl-2 family proteins [11, 16]. Bcl-2 family proteins modulation and MMP disruption are the characteristic features of mitochondrial apoptosis. Induction of the mitochondrial apoptotic route results in cytochrome C release which leads to the activation of caspase-9 and ultimately caspase-3. Caspase-3, being the main executioner of apoptotic machinery, cleaves PARP and other effector proteins, ultimately leading to apoptotic cell death [16, 18]. In line with established features of mitochondrial apoptosis, STM modulated Bcl-2 family proteins, dissipated MMP, and induced caspase-3 activation and cleavage of PARP in A549 lung cancer cells in a dose-dependent manner.

Nuclear factor-*κ*B (NF-*κ*B) is an extracellular signal-activated transcription factor that regulates the expression of various genes involved in cytokine production, cell proliferation and survival, drug resistance, and inflammatory processes. It is activated by a wide range of stimuli including cytokines, growth factors, carcinogens, and tumor promoters [24]. It is well established now that NF-*κ*B is constitutively overexpressed in various cancers including NSCLC [25]. Constitutive activation of NF-*κ*B is one of the major mechanisms of cancer cells to chemotherapy resistance [26]. The activation of NF-*κ*B is mainly induced by I*κ*B kinase (IKK) complex which is a component of the upstream NF-*κ*B signaling cascade. In normal resting cells, inhibitors of *κ*Bs (I*κ*Bs) bind with NF-*κ*B dimers and keep them in an inactive form in the cytosol. Upon ligand binding such as TNF-*α*, IKK becomes activated and in turn inhibits I*κ*B by inducing phosphorylation. Upon phosphorylation, I*κ*B dissociates from NF-*κ*B dimer which gets free and translocates into the nucleus [11]. Sesquiterpene lactones are well known for their anti-inflammatory activity and their proapoptotic activity has also been attributed to the inhibition of NF-*κ*B activation [11, 17]. Here, we found that STM inhibited the translocation of both constitutive and TNF-*α*-induced NF-*κ*B into the nucleus. Moreover, the inhibitory effect of STM on NF-*κ*B activation was found to be oxidative-stress-dependent. Moreover, STM inhibited the phosphorylation of I*κ*B-*α*, which might be one of the possible mechanisms of STM-induced inhibition of NF-*κ*B. However, further study is needed to investigate the detailed mechanism. IKK-16 treatment further increased the apoptotic effect of STM, indicating that inhibition of NF-*κ*B activation might be one of the mechanisms in STM-induced apoptosis.

**Figure 6:**
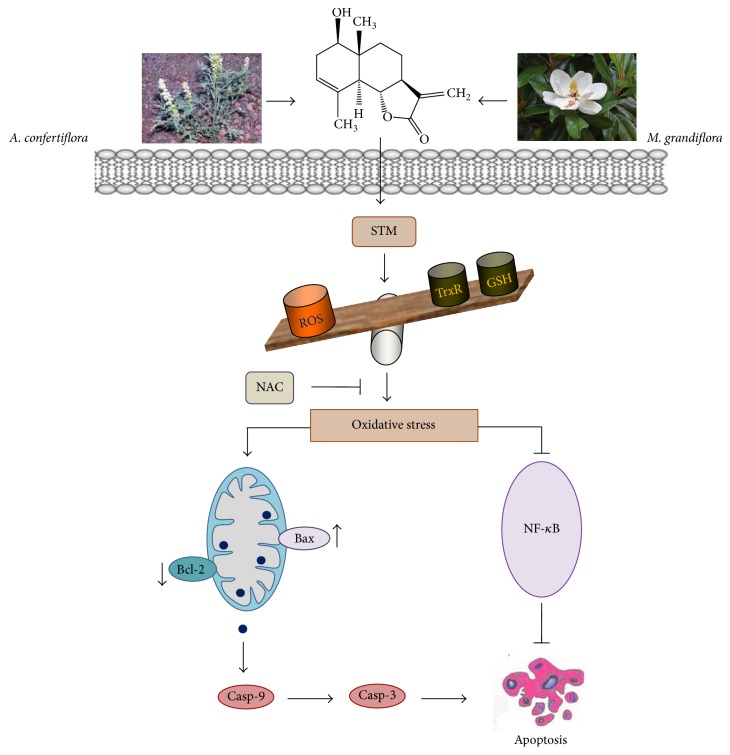
A schematic model of STM-induced apoptosis in A549 cells. STM induces oxidative stress by increasing ROS generation and GSH depletion and decreasing TrxR activity. STM-induced oxidative stress mediates apoptosis by inhibiting NF-*κ*B activation and inducing mitochondrial apoptotic pathways through Bax/Bcl-2 proteins modulation.

In conclusion, our data provided evidence for the first time that STM inhibits growth and induces oxidative stress, which results in NF-*κ*B inhibition, Bcl-2 family protein modulation, MMP dissipation, caspase-3 activation, and PARP cleavage, ultimately leading to mitochondrial apoptosis in A549 lung adenocarcinoma cells. Based on our experimental data, we have summarized a schematic model for the possible mechanism of STM-induced apoptosis in A549 cells as shown in Figure 6.

## References

[1] Roviello G. (2015). The distinctive nature of adenocarcinoma of the lung. *OncoTargets and Therapy*.

[2] Marshall A. L., Christiani D. C. (2013). Genetic susceptibility to lung cancer-light at the end of the tunnel?. *Carcinogenesis*.

[3] Nan J., Du Y., Chen X. (2014). TPCA-1 is a direct dual inhibitor of STAT3 and NF-*κ*B and regresses mutant EGFR-associated human non-small cell lung cancers. *Molecular Cancer Therapeutics*.

[4] Wu K., Chang Q., Lu Y. (2013). Gefitinib resistance resulted from STAT3-mediated Akt activation in lung cancer cells. *Oncotarget*.

[5] Castoldi R., Schanzer J., Panke C. (2016). TetraMabs: Simultaneous targeting of four oncogenic receptor tyrosine kinases for tumor growth inhibition in heterogeneous tumor cell populations. *Protein Engineering, Design and Selection*.

[6] Dong G., Mao Q., Xia W. (2016). PKM2 and cancer: the function of PKM2 beyond glycolysis. *Oncology Letters*.

[7] Li C., Zhang G., Zhao L., Ma Z., Chen H. (2016). Metabolic reprogramming in cancer cells: glycolysis, glutaminolysis, and Bcl-2 proteins as novel therapeutic targets for cancer. *World Journal of Surgical Oncology*.

[8] Khan M., Maryam A., Qazi J. I., Ma T. (2015). Targeting apoptosis and multiple signaling pathways with icariside II in cancer cells. *International Journal of Biological Sciences*.

[9] Khan M., Maryam A., Zhang H., Mehmood T., Ma T. (2016). Killing cancer with platycodin D through multiple mechanisms. *Journal of Cellular and Molecular Medicine*.

[10] Seo K. H., Ryu H. W., Park M. J. (2015). Mangosenone F, a furanoxanthone from garciana mangostana, induces reactive oxygen species-mediated apoptosis in lung cancer cells and decreases xenograft tumor growth. *Phytotherapy Research*.

[11] Mehmood T., Maryam A., Zhang H., Li Y., Khan M., Ma T. (2017). Deoxyelephantopin induces apoptosis in HepG2 cells via oxidative stress, NF-*κ*B inhibition and mitochondrial dysfunction. *BioFactors*.

[12] Li Y., Ni Z.-Y., Zhu M.-C. (2012). Antitumour activities of sesquiterpene lactones from inula helenium and inula japonica. *Zeitschrift für Naturforschung C*.

[13] El‐Feraly F. S., Chan Y. (1978). Isolation and characterization of the sesquiterpene lactones costunolide, parthenolide, costunolide diepoxide, santamarine, and reynosin from Magnolia grandiflora L. *Journal of Pharmaceutical Sciences*.

[14] Coronado-Aceves E. W., Velázquez C., Robles-Zepeda R. E. (2016). Reynosin and santamarine: two sesquiterpene lactones from ambrosia confertiflora with bactericidal activity against clinical strains of mycobacterium tuberculosis. *Pharmaceutical Biology*.

[15] Choi H.-G., Lee D.-S., Li B., Choi Y. H., Lee S.-H., Kim Y.-C. (2012). Santamarin, a sesquiterpene lactone isolated from Saussurea lappa, represses LPS-induced inflammatory responses via expression of heme oxygenase-1 in murine macrophage cells. *International Immunopharmacology*.

[16] Khan M., Li T., Ahmad Khan M. K. (2013). Alantolactone induces apoptosis in HepG2 cells through GSH depletion, inhibition of STAT3 activation, and mitochondrial dysfunction. *BioMed Research International*.

[17] Gach K., Janecka A. (2014). *α*-methylene-*γ*-lactones as a novel class of anti-leukemic agents. *Anti-Cancer Agents in Medicinal Chemistry*.

[18] Khan M., Ding C., Rasul A. (2012). Isoalantolactone induces reactive oxygen species mediated apoptosis in pancreatic carcinoma PANC-1 cells. *International Journal of Biological Sciences*.

[19] Chen W., Zou P., Zhao Z. (2016). Selective killing of gastric cancer cells by a small molecule via targeting TrxR1 and ROS-mediated ER stress activation. *Oncotarget*.

[20] Zou P., Xia Y., Ji J. (2016). Piperlongumine as a direct TrxR1 inhibitor with suppressive activity against gastric cancer. *Cancer Letters*.

[21] Ghantous A., Gali-Muhtasib H., Vuorela H., Saliba N. A., Darwiche N. (2010). What made sesquiterpene lactones reach cancer clinical trials?. *Drug Discovery Today*.

[22] Khan M., Yi F., Rasul A. (2012). Alantolactone induces apoptosis in glioblastoma cells via GSH depletion, ROS generation, and mitochondrial dysfunction. *IUBMB Life*.

[23] Zhang J., Li Y., Duan D., Yao J., Gao K., Fang J. (2016). Inhibition of thioredoxin reductase by alantolactone prompts oxidative stress-mediated apoptosis of HeLa cells. *Biochemical Pharmacology*.

[24] Chen W., Li Z., Bai L., Lin Y. (2011). NF-*κ*B in lung cancer, a carcinogenesis mediator and a prevention and therapy target. *Frontiers in Bioscience (Landmark Edition)*.

[25] Giopanou I., Lilis I., Papaleonidopoulos V. (2015). Comprehensive evaluation of nuclear factor-*κ*B expression patterns in non-small cell lung cancer. *PLoS ONE*.

[26] Körber M. I., Staribacher A., Ratzenböck I., Steger G., Mader R. M. (2016). NF-*κ*B-associated pathways in progression of chemoresistance to 5-fluorouracil in an in vitro model of colonic carcinoma. *Anticancer Research*.

